# Industrial Vegetable Oils: A Green Alternative for Enhancing Rubber Properties

**DOI:** 10.3390/polym17212898

**Published:** 2025-10-30

**Authors:** Julijana Žeravica, Olga Govedarica, Mirjana Jovičić, Sonja Stojanov, Dragan Govedarica

**Affiliations:** Faculty of Technology Novi Sad, University of Novi Sad, 21000 Novi Sad, Serbia; jzeravica@uns.ac.rs (J.Ž.); jovicicmirjana@uns.ac.rs (M.J.); sonja.stojanov@uns.ac.rs (S.S.); dragang@uns.ac.rs (D.G.)

**Keywords:** rubber, process oil, vegetable oil, hempseed oil, neural networks, natural rubber, rubber compounding

## Abstract

This study investigates the viability of industrial hempseed oil as a sustainable extender oil in rubber compounding, addressing the urgent demand for alternatives to petroleum-based oils due to regulatory pressures on polycyclic aromatic hydrocarbons (*PAH*). We employed automated neural networks to analyze the physical and mechanical properties of rubber composites containing industrial hempseed oil, comparing them with six vegetable oils and three petroleum-based oils at extender oil concentrations from 0 to 30 phr. The results revealed that compounds with 20 phr of industrial hempseed oil and raw soybean oil exhibited the highest cure rate index values of 64.32 1/min. Rubber samples with industrial hempseed oil showed a significant 18% reduction in hardness compared to conventional oils, with the softest rubber measuring 40.5 Shore A hardness at 30 phr. Additionally, energy consumption during mixing was decreased by up to 12% for vegetable oil samples compared to mineral oils, enhancing processing efficiency. The neural network approach yielded more accurate predictions of the cure rate index, Shore A hardness, and power consumption during rubber mixing, with a validation performance exceeding 99.2%. Sensitivity analysis identified key factors, including oil content and surface tension, influencing rubber hardness. Overall, this study underscores the potential of industrial hempseed oil as an effective, eco-friendly substitute for conventional mineral oils, contributing to more sustainable practices in the rubber industry.

## 1. Introduction

Process oils play a crucial role in the rubber industry, influencing the properties of natural rubber-based composites and enabling a wider range of rubber applications. To achieve the desired qualities in rubber products, it is essential to add rubber mixing components—particularly the type of process oil and reinforcing fillers—in precisely defined quantities. The selected process oil should enhance the processability of the rubber product, improve the dispersibility of the fillers within the rubber, and reduce the viscosity of the rubber mixture [1]. Consequently, carefully choosing the appropriate process oil can lead to reduced energy consumption during the rubber mixing phase.

Highly aromatic mineral oils have been favored in rubber compounding due to their compatibility with conventional elastomers [2]. However, increasing awareness of environmental and public health issues has led to the European Union’s *REACH* Regulation, which restricts the use of aromatic process oils with high levels of polycyclic aromatic hydrocarbons (*PAH*) [3,4]. In response, leading rubber manufacturers have begun to use eco-friendly process oils. It remains crucial to select mineral oils that can maintain the desired physical and mechanical properties of rubber products, even while meeting stringent *PAH* content requirements [3,5,6,7,8]. Numerous commercial mineral oils, such as Treated Distillate Aromatic Extract (*TDEA*), Mild Extract Solvates (*MES*), and naphthenic oils, can meet the low *PAH* content requirement of less than 3% by weight. However, because these oils have significantly different properties compared to highly aromatic oils, they may not be sufficiently compatible for producing optimal rubber mixtures [3]. An alternative to highly aromatic mineral oils is the use of vegetable oils and their derivatives [9].

Natural rubber elastomeric blends, known for their biodegradability, renewability, nontoxicity, and availability, are frequently researched in rubber compounding [1,10]. Careful consideration should be given to the selection and ratio of rubber components to create a product that meets market demands [1]. Many rubber companies are actively researching the development of green products based on renewable resources. Vegetable oils are recognized as a reliable alternative to petroleum-based extender oils in the rubber industry.

A variety of vegetable oils, including soybean oil, olive oil, orange peel oil, linseed oil, castor oil, palm oil, coconut oil, rice bran oil, rapeseed oil, hazelnut oil, rubber tree oil, cashew nut shell oil, jatropha oil, moringa oil, sunflower oil, neem oil and kurunj oil, are being explored as substitutes for conventional petroleum-based oils [2,7,11,12,13,14,15,16,17,18,19,20].

Additionally, modified vegetable oils such as epoxidized soybean oil, ozonized soybean oil, epoxidized palm oil, epoxidized hazelnut oil, epoxidized jatropha oil, and epoxidized neem oil have gained attention in industrial research due to their enhanced multifunctional performance [21,22,23].

Unlike mineral oils, vegetable oils are non-toxic, biodegradable, and readily available as renewable raw materials [9]. However, one of their disadvantages is oxidation stability, which tends to improve with saturated or monounsaturated fatty acids. As the unsaturation of triglycerides increases, their oxidation stability decreases, while low-temperature properties such as pour point and glass transition temperature improve [24]. The viscosity of vegetable oils is comparable to that of reference mineral extender oils. Moreover, vegetable oils typically have a viscosity index (*VI*) above 200, indicating only slight changes in viscosity with temperature variations, unlike mineral oils, where temperature has a significant impact on viscosity [24].

This study examines industrial hempseed oil as a promising vegetable extender oil for industrial applications. To our knowledge, there are currently no published studies analyzing the use of industrial hempseed oil in rubber compounding. Investigations have been conducted on the application of hempseed oil in fields such as biodegradable plastics, the construction sector, biofuels, and biodiesel [25,26,27,28]; however, its potential in natural rubber compounding remains unexplored. Interest in industrial hemp is increasing due to its numerous applications and environmental adaptability.

Recent studies show that vegetable and modified vegetable oils can match or improve key processing and mechanical performance metrics, such as filler dispersion, plasticization, curing behavior, and tensile/elongation properties. Additionally, they offer substantially lower *PAH* risks and better biodegradability [29,30,31,32,33].

Although vegetable extender oils have been studied for their potential to improve various rubber properties, there has been no systematic investigation of hempseed oil specifically as a rubber processing oil. As mentioned earlier, determining the type and quantity of components for the rubber compounding process is a critical issue. Improper selection of materials can delay rubber production, lead to various issues, and ultimately result in ineffective outcomes [34]. While various statistical techniques exist to optimize the rubber mixing process, some models may not be entirely reliable due to the nonlinear behavior of components involved in rubber compounding. Moreover, statistical approaches cannot accurately estimate the properties of rubber and compounding materials without laboratory testing.

Dasgupta et al. found that soybean-based processing aids can replicate many functional effects of petroleum oils while also reducing environmental impact [2]. Petrović et al. reported that soybean oil plasticizers enhance tensile strength and elongation in rubber formulations [11]. Jayewardhana et al. observed improved filler dispersion and curing behavior when using natural oils like linseed and castor oil as processing aids [12]. Xu et al. evaluated bio-based plasticizers derived from soybean oil for tire tread applications and identified improvements in elongation and reduced hardness for selected formulations [14]. Scarton et al. compared natural and epoxidized vegetable oils in elastomeric compositions, demonstrating that natural vegetable oils and epoxidized versions can enhance mechanical performance in tread rubber systems [18]. Rahman et al. showed that bio-based plasticizers can improve silica dispersion and mechanical strength in filled rubber compounds, supporting the industrial viability of modified vegetable oils [21]. Sarma et al. further emphasized the multifunctional benefits of epoxidized vegetable oils in enhancing filler-matrix interactions and overall compound performance [22].

Published findings consistently indicate that vegetable and modified vegetable oils can enhance filler wetting and dispersion, increase elongation at break and, in many cases, improve tensile performance compared to certain petroleum-derived oils, and reduce mixing energy when the physicochemical properties of the oils are favorable. Key oil descriptors identified in the literature include kinematic viscosity, solubility-parameter matching, surface tension, iodine number, and relative density. These descriptors significantly impact curing kinetics, hardness, and energy consumption during mixing [9,21,22].

Despite these advancements, industrial hempseed oil has not been systematically evaluated as a process oil in natural rubber compounding. Previous research on hempseed oil has primarily focused on biodegradable plastics, biofuels, and related industrial applications [25,26,27,28]. Consequently, the current literature lacks direct experimental comparisons of hempseed oil with representative vegetable and low-*PAH* mineral oils in a controlled natural rubber formulation and experimental modeling studies that identify which oil physicochemical descriptors most strongly influence cure rate, hardness, and mixing energy under consistent mixing conditions.

This study aims to address these gaps by characterizing the relevant physicochemical properties of industrial hempseed oil for rubber compounding, experimentally comparing the processing behavior and vulcanizate properties of formulations containing hempseed oil against six vegetable oils and three mineral oils, and applying artificial neural networks and global sensitivity analysis to predict cure rate index, Shore A hardness, and mixing power consumption. This will also rank the most influential oil parameters to provide guidance for formulation.

Artificial intelligence methods, especially supervised machine learning models such as artificial neural networks (*ANNs*), have proven to be valuable tools for formulating materials and optimizing processes in polymer science. The literature shows that *ANNs* can effectively capture complex, nonlinear relationships among various formulation variables, processing conditions, and the final properties of materials—something that traditional linear models struggle to achieve [35,36]. By using artificial neural networks, researchers can rapidly screen different formulations and generate response surfaces to aid in decision-making.

Artificial neural networks present a promising method for evaluating rubber characteristics, such as process control, system modeling, and several applications. Inspired by biological neural structures, *ANNs* are designed as simple systems that facilitate real-time decision-making. Their self-learning and self-adapting capabilities, along with their ability to model nonlinear behavior, make them particularly suitable for predicting the performance of rubber compounds [34,35,36,37,38]. At present, there is a lack of comprehensive research examining the use of industrial hempseed oil in the compounding of natural rubber. Specifically, there has been no integration of experimental characterization with sensitivity analysis driven by artificial neural networks.

This study will aim to assess the impact of the type and quantity of hempseed oil on the mixing parameters and properties of natural rubber in order to achieve the desired characteristics in finished natural rubber products. The properties of hempseed oil as a processing oil will be determined either through experimental methods or by calculating values. In the laboratory, internal batch mixers will be used to blend natural rubber at a constant temperature of 90 °C and a rotor speed of 60 rpm. Results will be compared with those of rubber compounds made using commercially available mineral and vegetable extender oils. A suitable mathematical model based on artificial neural networks will be employed to predict power consumption during the mixing phase, as well as various rubber properties, including cure rate index, hardness, elongation at break, modulus at 100% and 300%, and tensile strength.

## 2. Materials and Methods

### 2.1. Materials

Natural rubber (*NR*) and the rubber compounds used in this study are typically employed in the rubber compounding process. The components for rubber mixing, as shown in Table 1 are given on the basis of a total of 100 parts of raw natural rubber.

Natural rubber (*NR*), commercial name SVR CV60, manufactured by Vietnam Rubber Group, Geruco (Phu Nhuan, Vietnam) was used as the initial precursor, carbon black (N330), industrial hempseed oil (Agrojedinstvo, Serbia), linseed oil (Beohemik, Serbia), raw rapeseed (Victoriaoil, Serbia), raw sunflower (Victoriaoil, Serbia), raw soybean (Victoriaoil, Serbia), degummed rapeseed (Victoriaoil, Serbia), refined sunflower (Victoriaoil, Serbia) were added to the rubber mixture. In addition to hempseed oil (VO1), three environmentally friendly mineral oils—residual aromatic extract (*RAE*), treated aromatic extract (*TRAE*), and naphthenic oil—along with six vegetable oils (VO2 to VO7) were used as extender oils for rubber compounding. The experiments were conducted over a wide range of extender oil concentrations (0–30 phr). The investigated extender oils used in this study are listed in Table 2.

### 2.2. Rubber Mixing Process

The experiments were carried out using an internal batch mixer, specifically the HAAKE Rheo-mix 600 equipped with the HAAKE Rheocord EU-5 (manufactured by Thermo Fisher Scientific, Germany). Both the mixing temperature and speed were maintained at 90 °C and 60 rpm, respectively. During the experiment, voltage and current measurements were taken to calculate the power consumption during the rubber mixing phase.

### 2.3. Properties of Extender (Hempseed) Oil

The properties of used oils that have been researched or evaluated experimentally using standard methods include relative density, kinematic viscosity, viscosity index, viscosity-gravity constant, mean molecular weight, surface tension, refractive index, and iodine number.

The relative density of hempseed oil was measured using an Anton Paar density meter (4500 M), while its kinematic viscosity was determined with a Cannon-Fenske viscometer within a temperature range of 20 to 100 °C. The viscosity index (*VI*) was calculated according to the ASTM D2270 standard [39] method, based on the kinematic viscosities measured at 40 °C and 100 °C. Additionally, the viscosity-gravity constant (*VGC*) was calculated according to the ASTM D2501 standard [40] method, using the relative density and viscosities at 40 °C and 100 °C. The mean molecular weight was determined using the ASTM D2502 standard [41] test method. The surface tension of extender oils was measured using the stalagmometer method.

A solubility parameter can indicate how well an extender oil is compatible with natural rubber. The fatty acid composition of vegetable oils was utilized to calculate the Hansen solubility parameters [42,43], while Cataldo et al.’s equations were employed to determine the Hansen solubility parameters for mineral oils based on their *PNA* content [32,33].

### 2.4. Properties of Rubber

The investigation focused on the properties of rubber compounds and vulcanized rubber, as well as the impact of different types, proportions, and compositions of mineral and vegetable extender oils. The hardness of the rubber was measured using a durometer (*Shore hardness A*) in accordance with the ISO 7619-1 standard [44] test method. The effect of extender oil on the tensile strength (*T_s_)* of rubber products was evaluated at room temperature, following the ASTM D412 standard [45]. The ultimate tensile strength refers to the stress at which the material fractures, while the corresponding strain is defined as the elongation at break (*E_b_*).

Additionally, the study calculated various parameters, including the torque difference (*M*), modulus at 100% and 300% elongation, vulcanization speed (*V_c_*), the start time of the rubber compound vulcanization (*t_s_*_2_), and the optimal vulcanization time (*t*_90_).

### 2.5. Determination of Power Consumption

Power consumption during the effective mixing phase was determined from measured voltage *V* and current *I*.

### 2.6. Statistical Analysis

The statistical analysis was conducted on each natural rubber compound with varying amounts of extender oils, and the experimental data was fitted accordingly. Statistical analysis was carried out using the *TIBCO* Statistica Software Inc. (2020) and Data Science Workbench, version 14.

### 2.7. Artificial Neural Networks

Artificial neural networks (*ANNs*) have recently found applications in various scientific and technological fields, including polymer and elastomer research [46]. These networks are composed of multiple layers of small processing units called neurons, which learn iteratively from input data. Each neuron can receive one or more input signals without needing prior knowledge of the underlying functional relationships. These inputs are assigned weights and processed—typically using a nonlinear activation function—to produce an output signal. This paper provides new insights into the use of artificial neural networks for evaluating vegetable oils as extender oils.

Random sampling was employed as the sampling method, allocating 70% of the data for training, 15% for testing, and 15% for validation. Additionally, 1000 random seeds were used during the sampling process. The number of hidden layers in the neural network varied from 4 to 12. A total of 1000 networks were trained and evaluated using the sum of squares as the error function. The five neural networks with the lowest error scores (sum of squares) were retained for further analysis. In this study, an artificial neural network architecture consisting of three layers—input, hidden, and output—was used. Each computational unit (neuron) receives signals from other units or directly from the external environment through weighted connections (synapses). These incoming signals are adjusted by the synaptic weights, which either amplify or diminish the information based on their values [47].

Artificial neural networks were selected and trained using the Multilayer Perceptron (*MLP*) architecture, which is one of the most widely applied neural network architectures in materials modeling and process optimization. Broyden–Fletcher–Goldfarb–Shanno (*BFGS*) is a quasi-Newton second-order optimization method used for training networks, providing very fast convergence by approximating the Hessian matrix.

The input variables used for developing the Artificial Neural Network (*ANN*) included a set of physicochemical properties of extender oils and rubber mixtures, which are as follows:

- Fraction of extender oil in rubber mixture (phr);
- Kinematic viscosity at 90 °C (mm^2^/s);
- Surface tension at 90 °C (mN/m);
- Relative density at 90 °C;
- Mean molecular weight, kg/kmo;
- Refractive index;
- Hansen solubility parameter;
- Iodine number.

To enhance model training efficiency and ensure numerical stability, both the input and output datasets were normalized prior to analysis.

To calculate the relative influence (*RI*) of each input variable on key rubber performance metrics—specifically cure rate index, Shore A hardness, and power consumption during the rubber mixing process —a global sensitivity analysis was conducted using Statistica’s Automated Neural Networks module. This analysis employs a perturbation method, where the *ANN* model is retrained multiple times, each time excluding a different predictor variable. For each reduced model (with one predictor removed), performance degradation is assessed using metrics such as the sum of squares of residuals for regression. The relative importance of each variable is then calculated as:

(1) $${RI}_{i}=\frac{{SSR}_{reduced\left(i\right)}}{{SSR}_{full}}$$

where ${SSR}_{full}$ denotes the total sum of squared residuals for the complete model and ${SSR}_{reduced\left(i\right)}$ represents the same metric for the model without predictor *i*.

A higher *RI* value indicates a greater influence of the corresponding variable on model performance. These results were used to rank the predictors by importance, identifying the most critical physicochemical parameters that influence the rubber compounding process.

To ensure reproducible preprocessing and robust model evaluation, all inputs and outputs for the artificial neural networks were scaled using min-max normalization to fit within the range of [0, 1]. The scaling parameters, specifically the minimum and maximum values for each variable in the training set, were calculated solely from the training partition. These parameters were then applied to both the test and validation partitions to prevent any overlap of data.

Data were divided into training, test, and validation sets using repeated random sampling in a ratio of 70/15/15. Given the modest size of the experimental dataset, we aimed to reduce partitioning bias by generating splits with 1000 different random seeds. The retained partitions were chosen based on their ability to yield stable model selection statistics across the seeds. This approach enhanced reproducibility and minimized dependence on any single random split. Model development utilized Statistica’s Automated Neural Networks (*SANN*) multi-layer perceptron workflow. For each target response, we trained 1000 candidate *MLP*s, exploring hidden-layer sizes ranging from 4 to 12 neurons (each with a single hidden layer) and using various activation functions, including *identity*, *logistic*, *tanh*, and *exponential*. The training employed the Broyden–Fletcher–Goldfarb–Shanno (*BFGS*) quasi-Newton optimizer, with a maximum of 10,000 iterations, a convergence tolerance of 1∙10^−6^, and *L*2 weight decay (regularization) set to 1∙10^−4^. Early stopping was implemented based on an increase in validation sum-of-squares over five consecutive checks. For ensemble construction, we selected the five networks with the lowest validation sum-of-squares for each response. The reported performance metrics (*R*^2^ and root mean square error (*RMSE*) for training, test, and validation sets), as well as sensitivity analyses, were computed on the held-out validation set after inverse-scaling the predicted values back to their original units.

## 3. Results and Discussion

### 3.1. Rubber Process Oil Properties

The main physical and chemical characteristics of rubber process oil samples are presented in Table 3. The classification of these oils was conducted based on experimentally determined and calculated physico-chemical properties. Hempseed oil is recognized as a highly polyunsaturated oil, notably balanced with a ratio of 3:1 between two essential polyunsaturated fatty acids [25]. The predominant unsaturated fatty acids in hemp oil include palmitic acid (C16:0), stearic acid (C18:0), oleic acid (a monounsaturated fatty acid, C18:1), linoleic acid (a polyunsaturated fatty acid, C18:2), and alpha-linolenic acid (also a polyunsaturated fatty acid, C18:3). Hempseed oil has a total unsaturated fatty acid content of approximately 91%, which suggests that it is thermally unstable and prone to rapid oxidation. However, industrial hempseed oil may contain vitamins and natural antioxidants such as phytosterols, tocopherols, and phenolic compounds. These components help prolong the thermal stability and oxidation resistance of the unsaturated fatty acids, thereby enhancing the utilization of unsaturated bonds in the rubber compounding process [47].

The fatty acid composition of hempseed oil is presented in Table 4. The composition was determined according to EN ISO 12966 [48] using gas chromatography analysis.

Table 5 presents the calculated solubility parameters of six vegetable oils, labeled VO- 1 to VO-7, along with three mineral oils: *RAE*, *TRAE*, and naphthenic oil. The difference |Δ*δ*| between the solubility parameters of the extender oils and that of *NR* indicates the compatibility of the extender oil for rubber compounding. The results show that vegetable oils demonstrate greater compatibility with *NR* compared to mineral oils.

**Figure 1:**
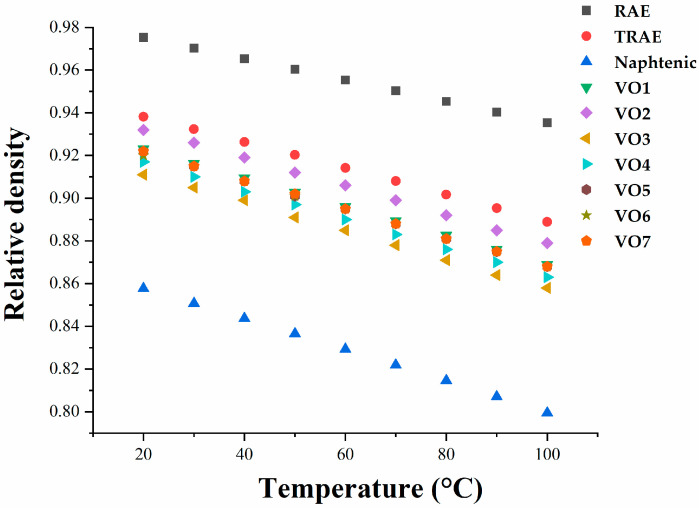
Dependence of the hempseed oil relative density on temperature compared to examined mineral and vegetable oils.

**Figure 2:**
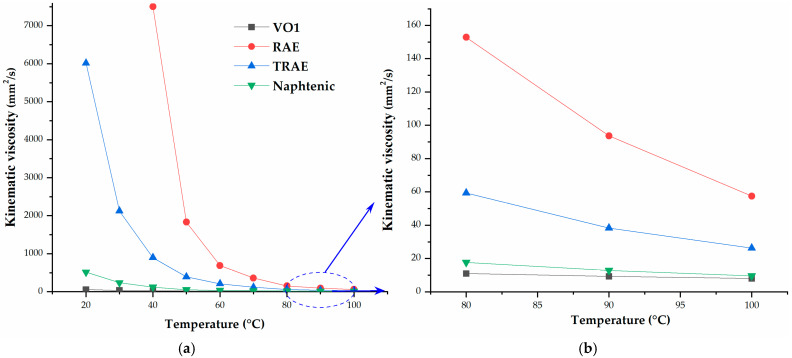
Kinematic viscosity versus temperature for hempseed oil and mineral oils, (**a**) across the entire temperature range; (**b**) from 80 to 100 °C.

**Figure 3:**
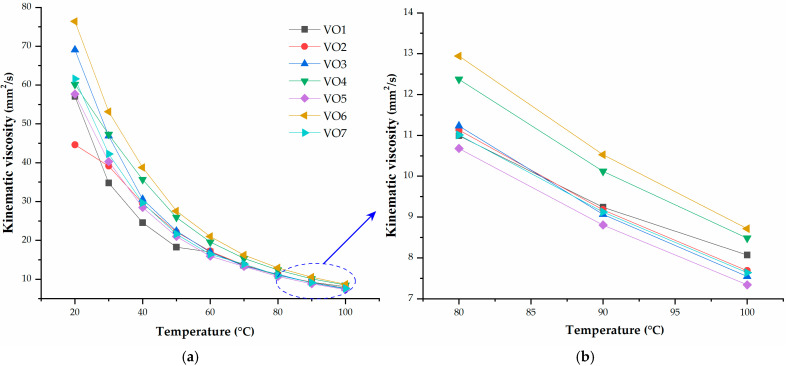
Dependence of the kinematic viscosity on temperature for vegetable oils, (**a**) across the entire temperature range; (**b**) from 80 to 100 °C.

The relative density and kinematic viscosity of all rubber process oils used in this study were significantly affected by temperature, as shown in Figure 1, Figure 2 and Figure 3, respectively. *RAE* and *TRAE* exhibited higher relative densities compared to hempseed oil, suggesting that hempseed oil may provide better dispersion of filler particles during the rubber mixing process [3]. The relative densities of the vegetable oils studied are comparable, with hempseed oil demonstrating a lower density than linseed oil.

Figure 2a indicates that hempseed oil has the lowest kinematic viscosity throughout the measured temperature range and experiences the smallest change in viscosity with temperature compared to the mineral reference oils. This behavior suggests that hempseed oil requires less energy for flow and maintains a flatter temperature-viscosity profile, which has practical implications for rubber compounding. The lower and more stable viscosity enhances oil mobility during rubber mixing. This promotes faster diffusion into the rubber matrix and more effective wetting of filler particles, aiding dispersion and reducing the overall compound viscosity. The reduced sensitivity to temperature fluctuations minimizes process variability, leading to more consistent mixing performance. This consistency simplifies the scale-up process from laboratory to pilot conditions. Extender oils with lower viscosity improve heat transfer and the transport of curatives during vulcanization, potentially increasing local reaction rates in certain composition ranges. From a formulation standpoint, the low viscosity of hempseed oil allows for the use of lower mixing temperatures or shorter high-shear stages while still achieving good dispersion.

*RAE* is the least suitable oil for the rubber compounding process due to its high kinematic viscosity. The differences in viscosity values are particularly noticeable at temperatures below 50 °C, where temperature significantly influences the kinematic viscosity of all the oils investigated. Among the examined oils, hempseed oil consistently exhibits the lowest viscosity across the entire temperature range and shows the least dependence on temperature compared to other mineral reference oils (Figure 2b). This characteristic suggests that rubber mixing can be conducted at lower temperatures with hempseed oil, allowing it to effectively diffuse between the rubber compounds during the mixing phase. In this paper, mixing was conducted at a temperature of 90 °C, and the kinematic viscosity of vegetable oils is further analyzed within this range (Figure 2b and Figure 3b). Only VO4 and VO6 exhibited higher kinematic viscosity in this temperature range.

Figure 3a illustrates the temperature dependence of kinematic viscosity across various vegetable oils. Compared to the mineral oils shown in Figure 2a, vegetable oils generally exhibit lower viscosities and narrower temperature-dependent ranges. Vegetable oils with similar low viscosities, such as hempseed and linseed oils, are likely to offer comparable processing advantages, including improved filler wetting and reduced mixing torque. In contrast, vegetable oils with higher viscosities (like VO4 and VO6 in this study) may act more like heavy extenders, potentially hindering curative mobility or necessitating longer mixing times. The variation in kinematic viscosity among vegetable oils suggests that not all vegetable oils are interchangeable. Therefore, selecting the appropriate vegetable oil should consider the intended processing conditions and desired final properties. The differences in viscosity stem from factors such as fatty acid composition and triglyceride structure and vegetable oils with higher degrees of unsaturation and shorter average chain lengths can tend to have lower viscosities and increased fluidity.

For practical applications, low-viscosity vegetable oils are preferred for energy-efficient mixing. However, to mitigate oxidation risks and minimize potential chemical interactions with curatives, it may be necessary to use antioxidants or blend them with less unsaturated oils for long-term stability.

**Figure 4:**
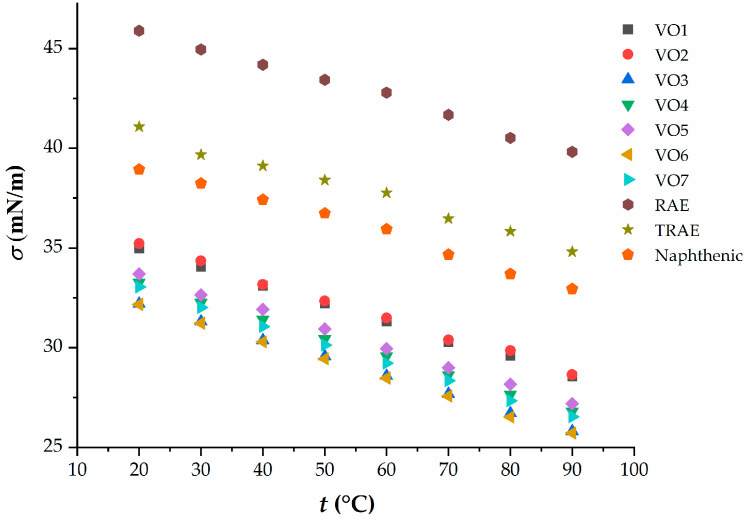
Dependence of the surface tension on temperature of vegetable and mineral oils.

Figure 4 illustrates the relationship between surface tension and temperature for hempseed oil compared to mineral and other vegetable oils.

Hempseed oil and the other vegetable oils studied have significantly lower surface tension compared to mineral oils. The addition of these oils enhances the dispersion of components during the rubber mixing process, resulting in lower energy consumption. The surface tension values of hempseed oil are comparable to those of linseed oil.

### 3.2. Effect of Oil Content and Nature on Rubber Properties

**Figure 5:**
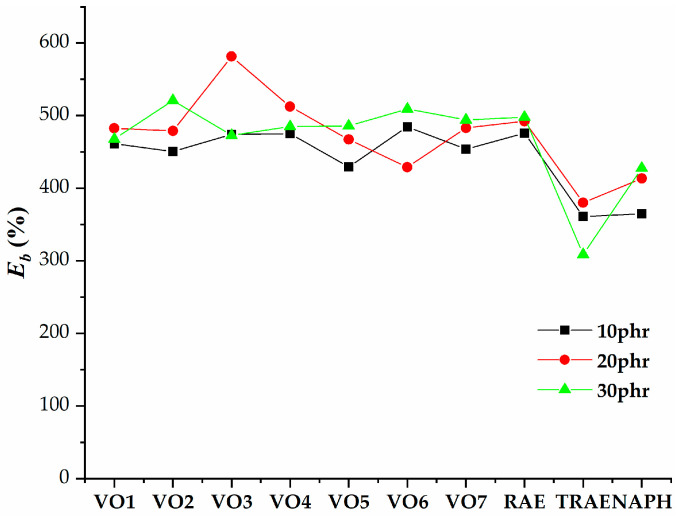
Dependence of the elongation at break of natural rubber (*NR*) compounds with varying amounts of extender oils.

**Figure 6:**
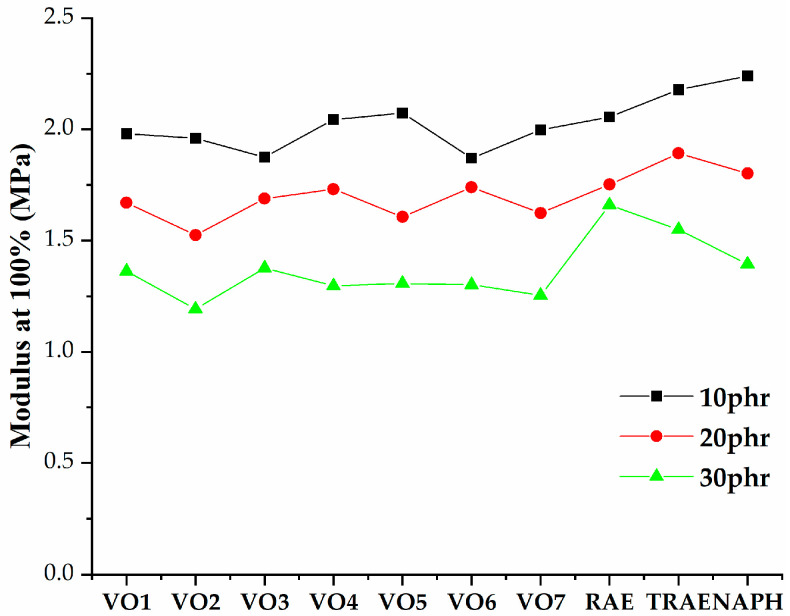
Modulus at 100% of natural rubber (*NR*) compounds with varying amounts of extender oils.

**Figure 7:**
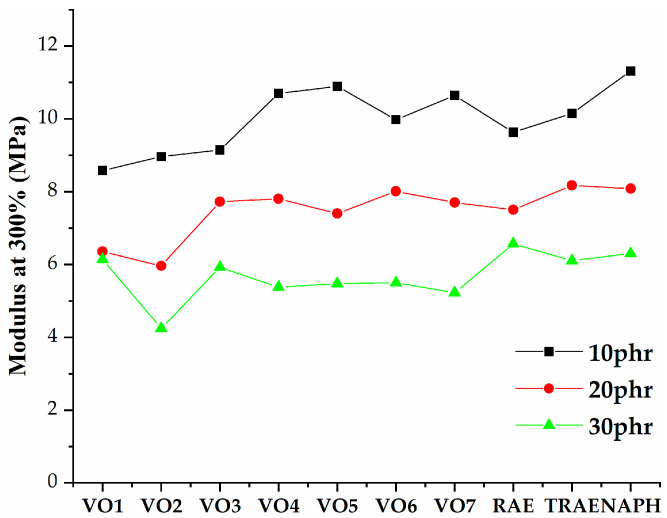
Modulus at 300% of natural rubber (*NR*) compounds with varying amounts of extender oils.

**Figure 8:**
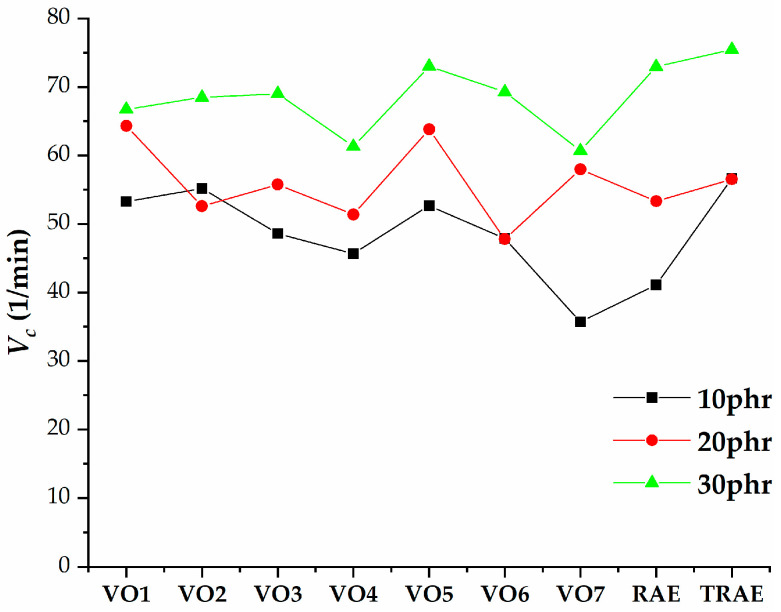
Cure rate index of natural rubber (*NR*) compounds with varying amounts of extender oils (phr).

**Figure 9:**
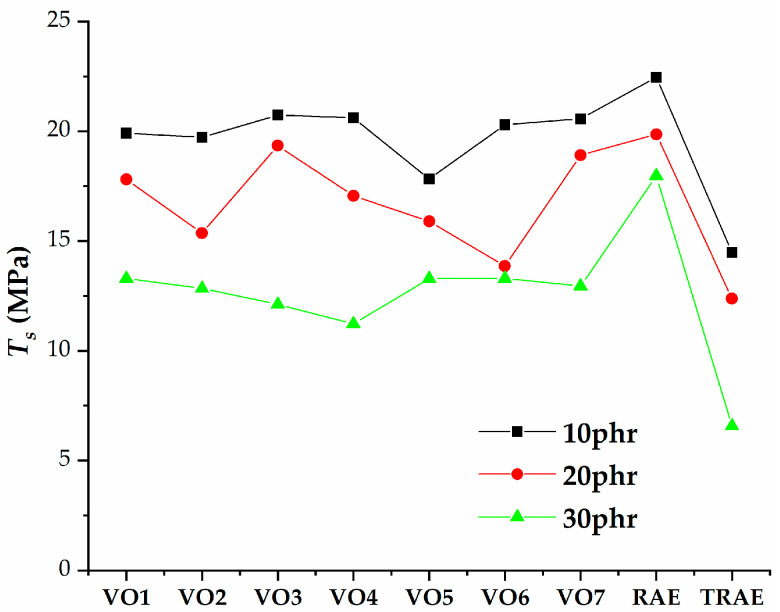
Tensile strength of natural rubber (*NR*) compounds with varying amounts of extender oils (*Ts*).

**Figure 10:**
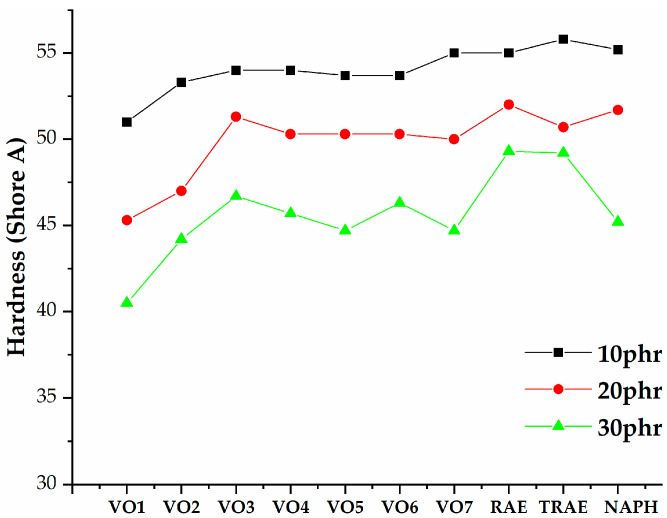
Dependence of the rubber hardness on the extender oil content.

**Figure 11:**
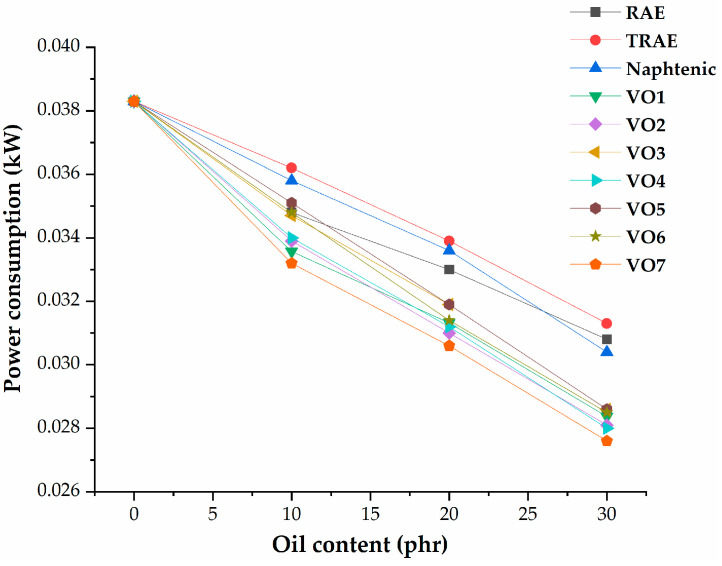
Dependence of the power consumption on the extender oil content.

Figure 5, Figure 6, Figure 7, Figure 8, Figure 9, Figure 10 and Figure 11 illustrate how oil content affects various properties such as elongation at break, modulus at 100% and 300%, curing speed index, tensile strength, hardness, and power consumption. It is evident that the elongation at break values for all vegetable oils exceed those obtained for *TRAE* and naphthenic oil, as shown in Figure 5.

The sample containing VO3 with an oil content of 20 phr showed the highest elongation at break, whereas the sample with *TRAE* and an oil content of 30 phr exhibited the lowest elongation at break. For hempseed oil, as well as VO4, VO7, and *RAE*, there was a relatively weak relationship between elongation at break and oil content, with values changing only slightly as the oil content increased.

Figure 6 and Figure 7 demonstrate that the tensile modulus decreases at both 100% and 300% with increasing oil content. For hempseed oil, the effect of oil content on the modulus at 300% becomes negligible once the oil content exceeds 20 phr. Samples that contained VO2 with an oil content of 30 phr exhibited the lowest values for both the 100% and 300% moduli. In contrast, samples with naphthenic oil at an oil content of 10 phr reached the highest modulus values.

Figure 8 illustrates that the cure rate index increases as the amount of extender oil increases, with the exception of samples containing VO2, VO6, and *TRAE* at an oil content of 20 phr. Rubber compounds with 10 phr of hempseed oil cured slightly more slowly than those with VO1 and *TRAE*, but faster than those with other reference oils. For the 20 phr oil content, compounds containing hempseed oil and VO5 exhibited the highest cure rate index values. At 30 phr, the highest values were observed in samples with *TRAE*, where hempseed oil samples cured slightly slower compared to most reference oils. The lowest cure rate index value was recorded for the sample with VO7 and an oil content of 10 phr.

Figure 9 shows that as the oil content increases, the tensile strength of the rubber samples decreases for all types of oil used. The rubber compounds that included vegetable extender oils exhibited higher tensile strength compared to those containing *TRAE* and naphthenic petroleum oil. The lowest tensile strength was observed in the sample with *TRAE* at an oil content of 30 phr.

The relationship between rubber hardness (Shore hardness A) and the content of hempseed oil, in comparison to reference oils, is illustrated in Figure 10. It was observed that increasing the amount of extender oil reduces the hardness of all tested rubber samples. Among the samples, those with hempseed oil consistently showed the lowest hardness at every level of oil content. The softest rubber was achieved with the maximum extender oil amount of 30 phr. Conversely, when only the minimum extender oil amount of 10 phr was used, the hardness of the rubber with hempseed oil was significantly lower than that of the samples containing reference oils.

The consistently lower hardness values observed for the samples containing hempseed oil can be explained by the specific composition and physicochemical properties of this oil. Hempseed oil is rich in polyunsaturated fatty acids, which account for more than 80% of its total fatty acid content. The high degree of unsaturation leads to increased molecular flexibility and reduced intermolecular interactions within the rubber matrix [49].

Additionally, the solubility parameter of hempseed oil is relatively close to that of the rubber phase, promoting better compatibility and more homogeneous dispersion. This enhances its plasticizing efficiency, as the oil molecules can penetrate between polymer chains and increase the free volume, resulting in reduced stiffness and lower hardness [19]. Therefore, the softer behavior of hempseed-oil-modified rubber is primarily attributed to its high unsaturation level, favorable solubility matching, and strong internal plasticizing effect.

The relationship between power consumption during the mixing phase of the rubber compounding process and oil content is illustrated in Figure 11. As the extender oil content increases, power consumption decreases for all types of oils used. Notably, samples containing vegetable oils exhibited lower energy consumption at each level of oil content compared to those with mineral oils, except for the sample with *RAE*, which showed lower consumption at an oil content of 10 phr.

Descriptive statistics of the mechanical and rheological properties of rubber are provided in Table 6.

### 3.3. Neural Networks and Global Sensitivity Analysis

Artificial neural networks were used in conjunction with global sensitivity analysis to predict the relative influence (*RI*) of input variables on rubber properties. The properties analyzed included power consumption, cure rate index, and rubber hardness during the rubber compounding process. A set of 21 samples obtained using vegetable oils was utilized to develop the neural networks. The Multilayer Perceptron (*MLP*) methodology was applied, featuring a minimum of 4 and a maximum of 12 hidden layers. A total of 1000 different neural networks were trained for each rubber property, and 5 networks were selected based on the mean square error function and the minimal sum of squares. The following activation functions were employed for the hidden and output neurons: *Identity*, *Logistic*, *Hyperbolic Tangent (Tanh)*, and *Exponential*.

Global sensitivity analysis was performed utilizing a perturbation re-training approach, whereby each predictor was systematically removed and the model was re-trained. The relative influence of each predictor was defined as the ratio of the validation sum of squares from the reduced model to that of the full model. Predictors were subsequently ranked by their relative influence, allowing for the identification of the most significant physico-chemical factors for each target response. Selected ensembles of artificial neural networks were employed to generate three-dimensional response surfaces that illustrate the interactions between the primary predictors and the modeled responses.

The analysis of *ANNs* yielded four distinct and innovative outputs pertinent to formulation and process decision-making: validated *ANNs* ensembles for three critical responses, accompanied by reported *R*^2^ and *RMSE* statistics; ranked lists of input variables based on relative influence, which elucidate the extender oil properties that most significantly affect cure rate, hardness, and power consumption; three-dimensional response surfaces that translate model behavior into specific ranges of oil properties and oil content, thereby facilitating the targeting of desired rubber outcomes; and uncertainty estimates derived from ensemble variability to enhance confidence in predictions. These outputs serve to provide actionable recommendations for the selection of environmental friendly extender oils and the determination of oil content ranges aimed at achieving softer rubber or minimizing power consumption during mixing phase.

#### 3.3.1. Cure Rate Index

**Figure 12:**
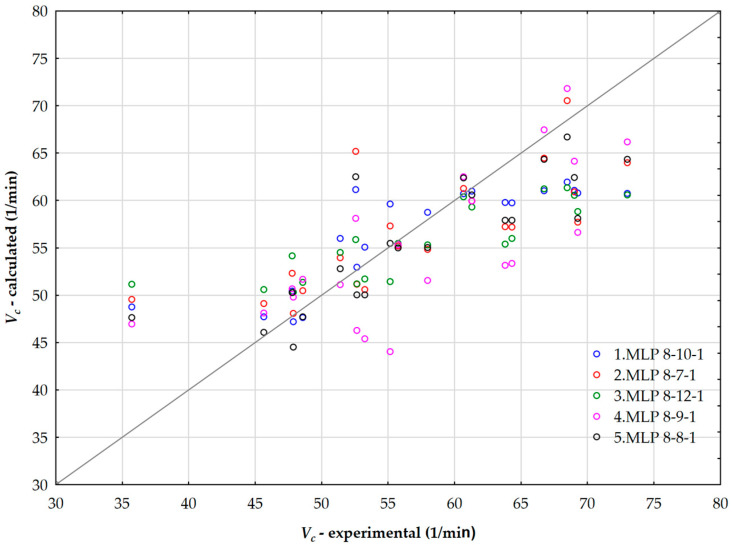
Parity plot of measured and calculated values of a cure rate index using ANN.

Table 7 presents a summary of the retained active neural networks used for calculating the cure rate index of rubber, along with details about the training algorithm, hidden layer, and output activation function. The neural network *MLP* 8-8-1 corresponds to architecture of 8 input layers, 8 hidden layers and 1 output layer. The highest validation performance of 0.999875 is observed for hyperbolic tangent hidden activation function, and logistic output activation. The selected active neural networks demonstrated comparable reliability and precision, with the highest validation performance achieved by the NN5 neural network. Figure 12 illustrates the relationship between predicted values of the cure rate index obtained through artificial neural networks and the values determined experimentally.

Based on a global sensitivity analysis of selected neural networks, the most influential parameters affecting the cure rate index were identified as oil content (phr), kinematic viscosity, and relative density.

**Figure 13:**
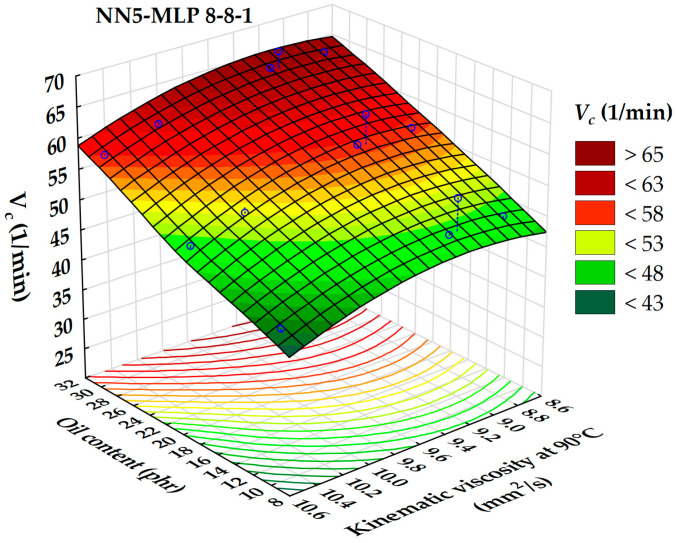
3D dependence of the cure rate index on oil content and kinematic viscosity.

The relationship between the cure rate index (*V_c_*), kinematic viscosity, and extender oil content is illustrated by a 3D surface in Figure 13. Figure 13 shows that an increase in oil content and a decrease in the oil’s kinematic viscosity result in higher *V_c_* values. This finding is consistent with previous research [50,51]. A local maximum for the cure rate index is observed within the kinematic viscosity range of 8.6 to 9.4 mm^2^/s. In contrast, the lowest cure rate index values are associated with oils that have a kinematic viscosity exceeding 10 mm^2^/s and oil contents below 12 phr. It is important to note that the content of extender oil has a more significant impact on the cure rate index than kinematic viscosity.

The dependence of the cure rate index on relative density at 90 °C, along with the content of the extender oil, was analyzed and presented as a three-dimensional surface in Figure 13. An increase in oil content and relative density results in higher cure rate index values. The lowest cure rate index values are recorded when the relative density is between 0.862 and 0.872, with oil content below 10 phr. Conversely, the highest cure rate index values are associated with relative densities exceeding 0.882 and oil contents above 30 phr. This finding reinforces the literature’s assertion that oil content has a greater influence on the cure rate index than relative density [50,51].

**Figure 14:**
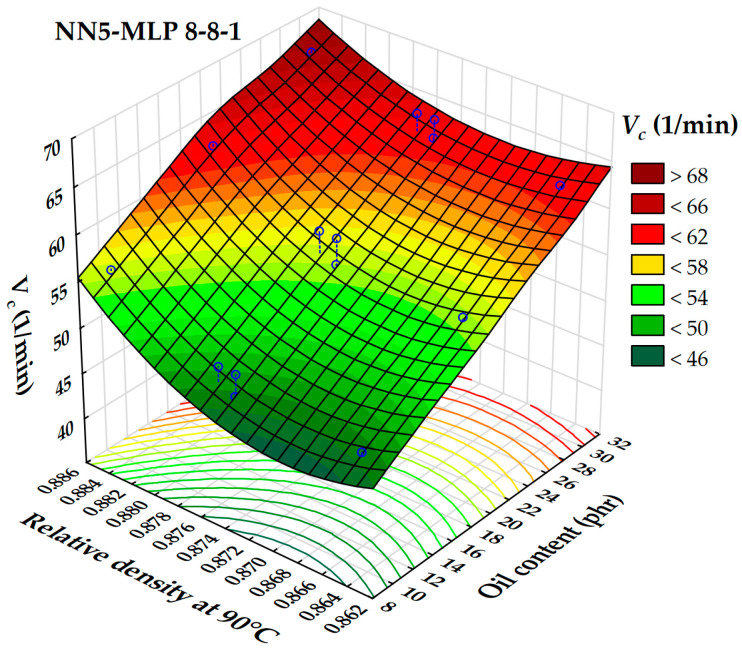
3D dependence of the cure rate index on oil content and relative density at 90 °C.

Figure 14 illustrates the dependence of the cure rate index (*V_c_)* based on the extender oil content (phr) and the extender oil’s relative density at 90 °C. The oil content has a significant influence on the cure rate index, with *V_c_* increasing consistently as the oil content rises across most relative density values. For any given oil content, a higher relative density is associated with a higher *V_c_*. Mechanistically, increasing oil content dilutes polymer–polymer interactions and plasticizes the rubber matrix, which enhances segmental mobility and accelerates the diffusion-limited steps of vulcanization (such as curative migration and chain encounters). This results in a higher observed cure rate index. Additionally, relative density likely serves as an indicator of oil composition (including aromaticity and molecular packing) and is correlated with curative solubility and heat capacity. Denser oils may solubilize curatives more effectively or improve local thermal properties, thereby speeding up the cure process. The 3D surface also reveals regions of low *V_c_* at low oil content combined with low-to-moderate densities, indicating that these formulations are susceptible to slow curing. Practical implications suggest that to increase the curing speed without altering curatives, one could either raise the oil content or select oils with a higher relative density. Conversely, to slow down curing for processing safety, one should reduce oil content or select lower-density oils.

It is important to note that the effect of density is secondary to oil content and may interact with viscosity and solubility parameters. Therefore, formulation choices should be experimentally validated, especially near processing or performance limits.

#### 3.3.2. Hardness

Table 8 shows a summary of the automated neural networks retained for calculating rubber hardness.

Retained neural networks have demonstrated similar reliability and precision, with the highest performance observed in the NN2 model (*MLP* 8-5-1). This neural network architecture consists of 8 input neurons, 5 hidden neurons, and 1 output neuron. The highest validation performance of 0.999999 was achieved using the hyperbolic tangent activation function for both the hidden and output layers.

Similar results were obtained with the NN3 model (*MLP* 8-7-1) and the NN5 model (*MLP* 8-4-1). However, NN3 employs logistic activation functions for both the hidden and output layers, while NN5 utilizes a logistic activation function in the hidden layer and an exponential activation function in the output layer.

**Figure 15:**
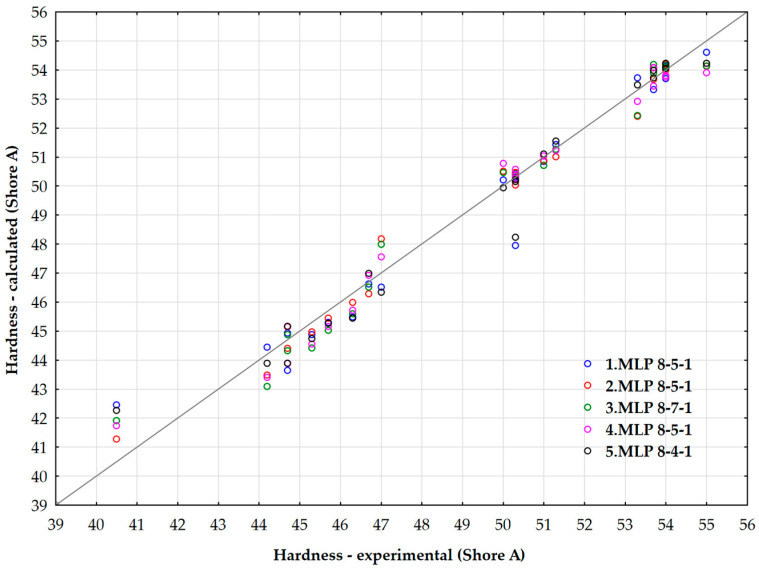
Parity plot of predicted values of a rubber hardness (Shore hardness A) using ANN.

Figure 15 illustrates the relationship between the predicted values of rubber hardness, as determined by the artificial neural networks (*ANNs*), and the experimentally measured rubber hardness values.

A global sensitivity analysis of selected neural networks identified several influential parameters affecting rubber hardness, including oil content (phr), surface tension, Hansen solubility parameters, mean molecular weight, and iodine number.

**Figure 16:**
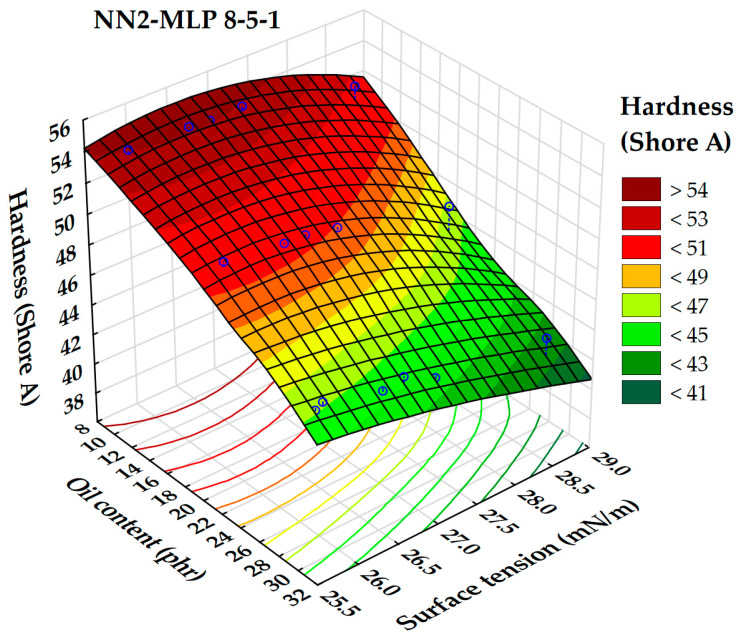
3D dependence of the rubber hardness (Shore hardness A) on oil content and surface tension.

The relationship between rubber hardness and both surface tension and extender oil content is illustrated by a 3D surface plot (Figure 16). The analysis shows that decreasing oil content and surface tension results in an increase in rubber hardness. Maximum hardness is observed when the oil content is below 12 phr and the surface tension is under 27 mN/m. This finding is consistent with previous research suggesting that lower oil content can lead to stiffer rubber compounds by reducing plasticization effects [52]. Furthermore, surface tension affects the mechanical properties of elastomers; lower surface energy typically results in increased hardness and improved mechanical performance in certain rubber systems. On the contrary, the lowest hardness values are seen when surface tension exceeds 28.5 mN/m and oil content exceeds 32 phr. This supports earlier research indicating that higher oil content and surface tension can produce softer, more flexible rubber materials [53].

**Figure 17:**
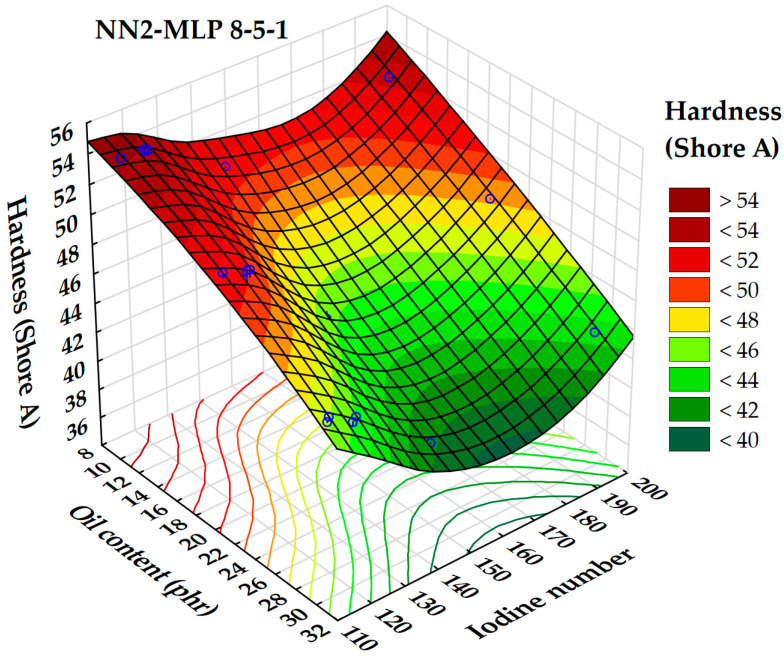
3D dependence of the rubber hardness (Shore hardness A) on oil content and iodine number.

The dependence between rubber hardness, iodine number, and oil content was modeled and represented as a 3D surface in Figure 17.

From Figure 17, the lowest rubber hardness values are observed when the oil content exceeds 30 phr across all ranges of iodine numbers. A local minimum in hardness occurs within the iodine number range of 150 to 170. Conversely, higher hardness values are achieved with oil contents below 18 phr and iodine numbers either below 120 or above 190. These findings are consistent with previous studies, which indicate that higher oil content can lead to increased plasticization, resulting in softer rubber compounds. Additionally, the iodine number, which measures the degree of unsaturation in oils, significantly influences the hardness of the rubber. Oils with higher iodine numbers, such as sunflower oil (iodine number 130), have been found to enhance the hardness and other physical properties of rubber compositions due to their ability to crosslink during the curing process.

#### 3.3.3. Power Consumption

Table 9 shows summary of retained active neural networks for calculation of power consumption during mixing phase

The selected automated neural networks exhibited similar reliability and precision, with NN4 (*MLP* 8-8-1) achieving the highest validation performance and the lowest standard validation error. The architecture of these neural networks demonstrates that both exponential and logistic activation functions can be effectively utilized in the hidden layer. For the output layer, hyperbolic tangent, exponential, logistic, and identity activation functions are applicable.

**Figure 18:**
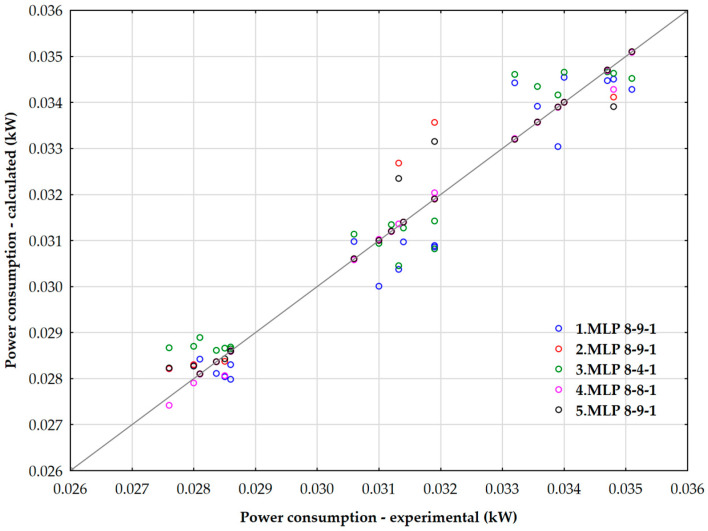
Parity plot of predicted values of power consumption during the mixing phase using an ANN.

Figure 18 shows the relationship between the predicted power consumption values using the artificial neural network (*ANN*) and the experimentally determined rubber consumption values.

According to a global sensitivity analysis of the selected neural networks, the factors that had the most significant impact on power consumption during the rubber compounding process were the iodine number, oil content (phr), Hansen solubility parameters, and surface tension.

**Figure 19:**
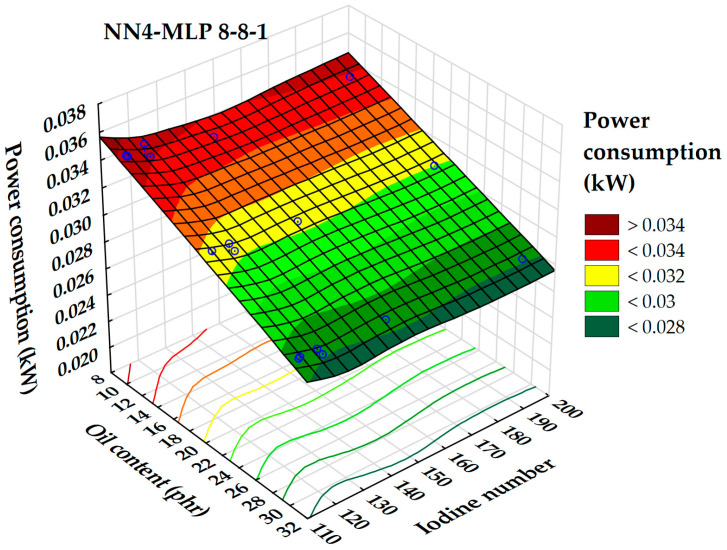
3D dependence of power consumption on oil content and iodine number.

Figure 19 illustrates the relationship between power consumption, iodine number, and extender oil content, represented in a 3D surface plot. It is clear that reducing the oil content across the entire range of iodine numbers leads to an increase in power consumption. Notably, power consumption remains lower when the oil content exceeds 30 parts per hundred rubber (phr) throughout the iodine number spectrum. The highest power consumption values occur when the oil content is below 10 phr, particularly at iodine numbers lower than 130 or higher than 180. This trend is similar to the dependency observed in rubber hardness under the same parameters. However, there are no distinct local maxima or minima within the examined range.

Generally, the interaction between extender oil composition, iodine value, and energy consumption is complex. By carefully selecting and optimizing these parameters, it is possible to improve energy efficiency and sustainability in rubber processing. Additionally, the choice of extender oil not only influences power consumption but also carries environmental implications. Transitioning to vegetable oils aligns with sustainability goals by reducing dependence on petroleum-based products. This shift can positively impact both the energy efficiency and environmental footprint of the rubber compounding process [19].

**Figure 20:**
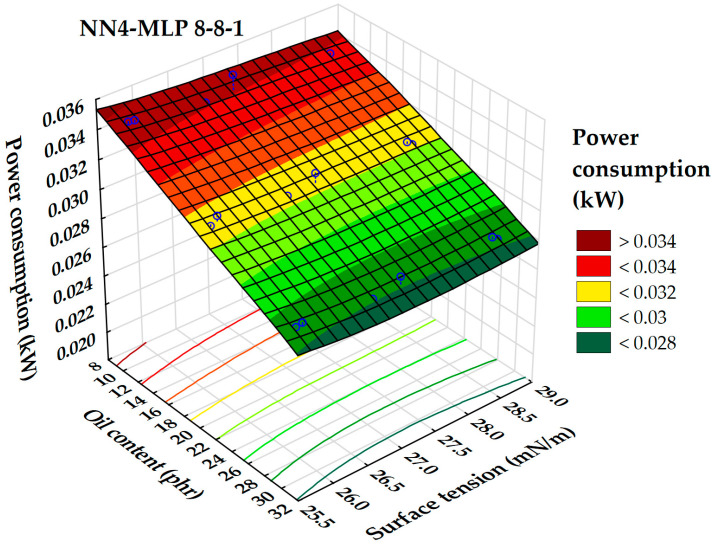
Three-dimensional dependence of power consumption on oil content and surface tension.

The relationship between power consumption, surface tension, and extender oil content is illustrated in a 3D surface plot (Figure 20). This analysis confirms that increasing oil content leads to higher power consumption. Notably, once the oil content exceeds 30 phr, power consumption levels off, with a small local minimum observed between 27 and 28 mN/m. The highest power consumption values during the mixing phase occur with oil contents below 10 phr and surface tensions under 26 mN/m. This indicates that both higher oil content and surface tension contribute to more efficient mixing processes.

This trend is consistent with the observed dependency in rubber hardness regarding these same parameters. The interaction between oil content and surface tension significantly affects power consumption during the rubber mixing process. By adjusting these parameters, it is possible to optimize energy efficiency and enhance the overall quality of the rubber product.

The following discussion links the measured extender oil descriptors (viscosity, surface tension, Hansen solubility parameter, iodine number, relative density, mean molecular weight) to the observed macroscopic responses (hardness, cure rate index, power consumption, tensile/elongation) using molecular interactions and thermodynamic interpretation of experimental trends and polymer-physics principles.

Extender oils that act as effective plasticizers increase free volume and polymer segmental mobility, lowering modulus and Shore A hardness. Plasticization strength depends on thermodynamic affinity (solubility parameter matching) and the ability of oil molecules to intercalate between polymer chains. Hempseed oil’s small |Δ*δ*| with *NR* and relatively low kinematic viscosity enable deeper penetration into the rubber network, increasing chain mobility and reducing hardness. Better miscibility (lower Gibbs free energy of mixing) arises when oil and rubber solubility parameters converge, promoting uptake of oil into amorphous rubber domains and amplifying plasticization.

Lower surface tension enhances wetting of carbon black and filler surfaces, improving filler–oil–polymer interfacial contact. Improved wetting reduces filler agglomeration, lowers compound viscosity, and decreases internal friction during mixing, which reduces measured power consumption. Better dispersion also yields more uniform stress transfer and often higher elongation at break and tensile performance for comparable stiffness. Interfacial free energies govern wetting and capillarity. Furthermore, lower oil–air and oil–solid interfacial tensions favor spreading on particle surfaces and thermodynamically driven deagglomeration.

Cure kinetics for sulfur vulcanization include diffusion-limited steps (curative migration, activator transport) and reaction-limited steps. Lower oil viscosity increases diffusion coefficients for small curative molecules and accelerates their homogenization in the matrix, increasing local reaction rates and observed cure rate index. Very low kinematic viscosity can dilute curatives excessively or change microenvironment polarity, producing a non-monotonic effect and explaining the observed local maximum. High viscosity oils hinder curative mobility and slow cure.

A high iodine number indicates greater unsaturation and potential chemical reactivity under vulcanization conditions. Unsaturated triglycerides can (a) act as secondary reactive sites contributing to network formation or (b) undergo competing oxidative or radical reactions that alter crosslink density. Both mechanisms change final modulus and hardness: reactive oils may increase crosslinking locally (raising hardness), while nonreactive but highly unsaturated oils primarily plasticize (lowering hardness). Hempseed oil’s behavior (softening) suggests dominant plasticization and physical compatibility effects in the tested formulations, possibly moderated by natural antioxidants that reduced unwanted side reactions.

Relative density often correlates with aromatic or heavier fractions in oils. Generally, denser oils may solubilize curatives differently or modify thermal capacity and heat transfer during mixing and cure. Mean molecular weight affects free volume contribution per oil molecule: lower molecular weight molecules more effectively plasticize per mass unit. In contrast, higher molecular weight species contribute less to chain mobility and may increase kinematic viscosity.

The cross-validation results for second-order polynomial regression (Table 10) were compared directly with the automated neural network ensembles developed in this study.

For the cure rate index, the polynomial model demonstrates limited predictive ability, with a predicted *R*^2^ of 0.6127. In contrast, the retained ANN ensemble achieves substantially higher validation performance, with *R*^2^ values reaching up to 0.999875 across selected networks. This indicates that the ANN captures the nonlinear cure kinetics much more effectively. For Shore A hardness, the polynomial model performs well, yielding a predicted *R*^2^ of 0.9318. However, it remains inferior to the ANN ensemble, which achieves nearly perfect validation *R*^2^ values of up to 0.999999 and exhibits lower dispersion. This demonstrates superior fit and predictive stability. In terms of power consumption, the polynomial baseline also provides strong results, with a predicted *R*^2^ of 0.9832. Nevertheless, it is again outperformed by the ANN models, which deliver virtually perfect validation performance, with an *R*^2^ close to 1.000000 and minimal standard error. Overall, while polynomial regression offers a valuable and interpretable baseline for hardness and power consumption, it falls short in robustly modeling cure rate behavior. The automated MLP ensembles consistently yield higher predictive accuracy and are the preferred modeling approach for this dataset.

From a life cycle perspective, vegetable oils such as hempseed oil offer notable environmental advantages over conventional mineral oils. *LCA* studies have shown that the global warming potential of hempseed oil production is 50–70% lower than that of petroleum-derived oils, mainly due to its renewable origin and carbon sequestration capacity during hemp cultivation. In addition, hemp cultivation requires limited pesticide use and supports soil regeneration, contributing to a more sustainable material profile. Therefore, the application of hempseed oil as a processing additive in rubber aligns with current trends toward bio-based and environmentally responsible materials [54,55,56].

## 4. Conclusions

This study presents the following key findings:

- Hempseed oil is an effective and environmentally friendly extender oil that enhances processability and produces softer natural rubber vulcanizates when compared to traditional mineral and vegetable oils.
- Compounds that contain vegetable extender oils require less mixing power than those made with mineral oils. This indicates improved filler dispersion and reduced compound viscosity.
- A global sensitivity analysis identified several key physico-chemical factors, such as oil content, surface tension, the Hansen solubility parameter, mean molecular weight, and iodine number, as the most influential predictors of the cure rate index, Shore A hardness, and power consumption during mixing.
- Ensembles of Multilayer Perceptron neural networks are capable of accurately predicting the cure rate index, Shore A hardness, and mixing power. They also provide actionable three-dimensional response surfaces that can guide formulation decisions.

## 5. Limitations and Future Work

Future research should include accelerated aging and extended oxidative stability studies for hempseed-oil formulations and blends with less unsaturated or modified vegetable oils. Expanding the experimental dataset to include additional oil chemistries, wider oil content ranges, and application-specific performance metrics is essential for subsequent retraining and external validation of *ANNs* ensembles. Moreover, exploring optimized oil blends, along with antioxidant and additive strategies, will help achieve a balance between softness, stability, and curing behavior for targeted rubber applications.

## Figures and Tables

**Table 1:** Natural rubber compounding formulations.

Ingredient	Function	Amount (phr)			
*NR*	Matrix	100	100	100	100
Extender oil	Processing aid/plasticizer	0	10	20	30
*ZnO*	Activator	5	5	5	5
Stearic acid	Activator/ dispersing aid	2	2	2	2
Carbon black	Reinforcing filler	30	30	30	30
*IPPD* *	Antioxidant	1	1	1	1
*CBS* *	Accelerator	2	2	2	2
Sulfur	Curing agent	2.5	2.5	2.5	2.5

* N-cyclohexyl-2-benzothiazole sulfonamide (*CBS*); N-isopropyl-N′-phenyl-N-cyclohexyl-2-benzothiazole sulfonamide p-phenylenediamine (*IPPD*).

**Table 2:** Vegetable and mineral oils examined in this study.

Vegetable Oils							Mineral Oils		
VO1	VO2	VO3	VO4	VO5	VO6	VO7	*RAE*	*TRAE*	*Naphthenic*
Hempseed	Linseed	Raw rapeseed	Raw sunflower	Raw soybean	Degummed rapeseed	Refined sunflower	Residual aromatic extract	Treated aromatic extract	*Naphthenic*

**Table 3:** Physical and chemical characteristics of vegetable oils.

	VO1	VO2	VO3	VO4	VO5	VO6	VO7
Relative density at 90 °C	0.87607	0.88550	0.86450	0.86970	0.87465	0.87467	0.87470
Kinematic viscosity at 90 °C, mm^2^/s	9.2422	8.8863	9.0119	9.9224	8.7658	10.4536	9.1206
*VI*	186.95	249	231	228	242	214	245
*VGC*	0.88825	0.901	0.872	0.881	0.887	0.882	0.886
Refraction index at 20 °C	1.4765	1.4802	1.4719	1.4735	1.4746	1.4715	1.4732
Iodine number (g of iodine/100 g of oil)	143.9	194.41	113.88	121.65	119.77	113.39	120.75
Mean molecular weight, kg/kmol	882.81	874.22	881.40	879.02	876.27	882.10	879.11
Surface tension, mN/m	28.56	28.64	25.81	26.80	27.20	25.72	26.54

**Table 4:** The fatty acid compositions of industrial hempseed oil.

Composition	VO1
Palmitic acid (C16:0), %	8.51
Stearic acid (C18:0), %	3.44
Oleic acid (C18:1), %	17.10
Linoleic acid (C18:2), %	51.18
γ-Linolenic acid (C18:3n6), %	3.18
α-linolenic (C18:3n3), %	13.30
Other fatty acids, %	3.29

**Table 5:** Determined Hansen solubility parameter, *δ_mixture,_* and compatibility of extender oils with *NR*, |Δ*δ*|.

Extender Oil	*δ_mixture_* (MPa^1/2^)	|Δ*δ| = |δ_NR_* − *δ_mixture_|*
VO1	16.8184	0.1184
VO2	16.731	0.0310
VO3	16.865	0.1650
VO4	16.869	0.1690
VO5	16.850	0.1500
VO6	16.850	0.1500
VO7	16.869	0.1690
*RAE*	15.716	0.9840
*TRAE*	15.803	0.8966
*Naphthenic*	15.028	1.6723

**Table 6:** Descriptive statistics of the mechanical and rheological properties of rubber.

Mechanical or Rheological Property	Standard Deviation	First Quartile	Median	Third Quartile	Mean Value
*Hardness* (Shore A)	±4.105	45.500	50.300	53.500	49.143
*V_c_* (1/min)	±9.54	49.99	55.75	65.53	57.19
*T_s_* (MPa)	±3.303	13.289	17.060	19.820	16.520
*E_b_* (%)	±32.91	464.15	479.03	490.00	480.90
*M*_100_ (MPa)	±0.2900	1.3348	1.6700	1.9176	1.6417
*M*_300_ (MPa)	±2.008	5.715	7.705	9.058	7.514
*Power consumption* (kW)	±0.002532	0.028550	0.031317	0.033735	0.031255

**Table 7:** Summary of active networks for calculation of cure rate index.

Index	Network Name	Training Performance	Test Performance	Validation Performance	Training Algorithm	Hidden Activation	Output Activation
NN1	*MLP* 8-10-1	0.822928	0.999747	0.994468	*BFGS* 2	*Logistic*	*Exponential*
NN2	*MLP* 8-7-1	0.788813	0.991634	0.992451	*BFGS* 5	*Exponential*	*Logistic*
NN3	*MLP* 8-12-1	0.869379	0.990547	0.997150	*BFGS* 3	*Exponential*	*Identity*
NN4	*MLP* 8-9-1	0.802732	0.973328	0.999599	*BFGS* 6	*Exponential*	*Logistic*
NN5	*MLP* 8-8-1	0.863681	0.991025	0.999875	*BFGS* 4	*Tanh*	*Logistic*

**Table 8:** Summary of active networks for calculation of hardness.

Index	Network Name	Training Performance	Test Performance	Validation Performance	Training Algorithm	Hidden Activation	Output Activation
NN1	*MLP* 8-5-1	0.988800	0.997974	0.999990	*BFGS* 27	*Tanh*	*Exponential*
NN2	*MLP* 8-5-1	0.990519	0.997300	0.999999	*BFGS* 17	*Tanh*	*Tanh*
NN3	*MLP* 8-7-1	0.987565	0.996226	0.999997	*BFGS* 33	*Logistic*	*Logistic*
NN4	*MLP* 8-5-1	0.989657	0.994869	0.999993	*BFGS* 16	*Tanh*	*Logistic*
NN5	*MLP* 8-4-1	0.989811	0.995998	0.999997	*BFGS* 27	*Logistic*	*Exponential*

**Table 9:** Summary of active networks for calculation of power consumption during mixing phase.

Index	Network Name	Training Performance	Test Performance	Validation Performance	Training Algorithm	Hidden Activation	Output Activation
NN1	*MLP* 8-9-1	0.966634	0.999264	0.999999	*BFGS* 4	*Exponential*	*Tanh*
NN2	*MLP* 8-9-1	0.999999	0.999351	1.000000	*BFGS* 1001	*Exponential*	*Exponential*
NN3	*MLP* 8-4-1	0.977186	0.998968	0.999998	*BFGS* 7	*Logistic*	*Logistic*
NN4	*MLP* 8-8-1	0.999985	0.999904	1.000000	*BFGS* 74	*Exponential*	*Identity*
NN5	*MLP* 8-9-1	0.999999	0.999091	0.999999	*BFGS* 2703	*Exponential*	*Exponential*

**Table 10:** Cross-validation with alternative regression models (polynomial regression, second order).

	*R* ^2^	*R*^2^ Adjusted	*R*^2^ Predicted	Standard Deviation
*V_c_* (1/min)	0.8464	0.7637	0.6127	4.63744
*Hardness* (Shore A)	0.9743	0.9605	0.9318	0.815879
*Power consumption* (kW)	0.9938	0.9905	0.9832	0.0002472

## Data Availability

The original contributions presented in this study are included in the article. Further inquiries can be directed to the corresponding author.
